# Medical students are not blank slates: Positionality and curriculum interact to develop professional identity

**DOI:** 10.1007/s40037-017-0402-9

**Published:** 2018-01-05

**Authors:** Kirkpatrick B. Fergus, Bronte Teale, Milani Sivapragasam, Omar Mesina, Erene Stergiopoulos

**Affiliations:** 1 School of Medicine, University of California—San Francisco, San Francisco, CA USA; 2 School of Medicine, Western Sydney University—Campbelltown, Sydney, Australia; 3 Faculty of Medicine, McGill University, Montreal, Quebec Canada; 4 Faculty of Medicine, University of Toronto, Toronto, Ontario Canada

As Stubbing et al. [1] have rightly pointed out, medical students do not enter their training as ‘blank slates’. We carry with us diverse life experiences, which influence early notions of what it means to be a doctor. As co-authors of this accompanying commentary, and as medical students from universities in the US, Canada, and Australia, we entered medical school with a range of preconceptions of what makes a doctor. In writing this commentary, we have drawn from our varied and unique perspectives, including our respective experiences as members of communities often underrepresented in medicine, including LGBT (lesbian, gay, bisexual and transgender), racialized, disabled, refugee, undocumented, and low socioeconomic status communities. Of course, we do not represent the communities to which we belong, and our composite identities include communities well-represented in medicine. Further, our lived experiences cannot possibly capture the multifarious backgrounds of physicians in training. However, these experiences have been influential in our trajectory—they guided our aspirations to pursue medicine as a career, and at times hindered our pathway to medicine, and continually shape our notion of what it means to be a physician. As a result, this commentary aims to reflect on how diverse medical student positionalities influence professional identity formation. Moreover, we aim to highlight the role of medical education institutions’ formal and hidden curricula in empowering students to develop a professional identity that embraces their positionality.

## Diversity matters

**Fig. 1:**
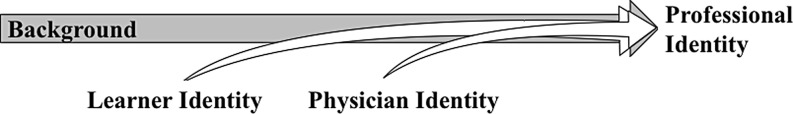
Integrating past and future: Supporting student backgrounds in professional identity formation

Medical schools internationally have embraced a mandate of social accountability, so that medical students may reflect the diverse populations they will serve [2, 3]. However, when these increasingly diverse medical students enter training, they are confronted with pressures to conform to a preset professional identity [4]. These pressures occur both at the level of formal curricular teaching, and through the hidden curriculum—that is, the institutional practices, policies, and language that shape learners’ perceptions of their student role [5]. Stubbing et al. rightly describe the learner identity as one important element of early professional identity formation [1]. As medical students, our pre-conceived ‘figured world’ is indeed at odds with the reality of medical training for a number of reasons, and supporting medical students through this tension is vital. However, we contend that learners’ lived experiences—in particular, the diverse identities we held before medical school—are essential to acknowledge as we develop our professional roles (Fig. 1). To illustrate this, we provide three thematic examples: privilege, stigma, and micro-aggressions.

How we self-identify in relation to privilege impacts our figured preconceptions of medical school. For example, while some students have physician parents or significant exposure to medicine, others enter medical school with limited knowledge about the profession. The social capital disparity is manifest in many communities, such as those of low socioeconomic status, racial and ethnic minorities, and rural upbringings. This disparity lingers throughout medical school, challenging these students’ preparedness for academic and clinical endeavours [6, 7]. At the same time, we find compelling what one medical student from an ‘underprivileged’ background wrote that her unique identity enhanced her ability to practice empathic care and understand the social determinants of health [8].

Learners with marginalized identities may also face significant stigma. For example, medical culture often frames illness as a weakness or failure to cope [9, 10]. As a result, medical students with disabilities often hesitate to disclose a disability out of fear of being scrutinized by classmates or teachers, or being judged as less competent [11]. Similarly, sexual and gender minorities face stigma, with one study finding that 29.5% concealed their identity for fear of discrimination [12]. Yet these learners hold valuable perspectives—for example, as recipients of healthcare and as self-advocates—which we translate into our own development as healthcare providers.

Beyond the effects of overt stigma, discrimination and harassment, learners commonly experience everyday slights and micro-aggressions in their educational and clinical placements [7, 13, 14]. In particular, we who identify as racial minorities must navigate this climate daily, which can adversely impact our wellbeing [15]. Our professional identity is fashioned by our sociocultural upbringing in parallel with our medical training, and many of us face the particulars of this ‘emotional tension’ without adequate support.

## Implications for curriculum development

How then can medical institutions support students to become physicians who honour their lived experiences? While the medical education community is making great efforts to address this issue, we draw on our experiences as students to suggest three key approaches institutions can adopt to better facilitate professional identity formation by meaningfully embracing diversity [16].

### Access to student services

Issues surrounding wellbeing (including discrimination, harassment and mental health) can disproportionately affect students from marginalized backgrounds [14, 17]. We urge medical institutions to provide appropriate accommodations (e. g. flexibility in scheduling), disability services [18], mental health and counselling services, well-being workshops, and adequate financial support. Making these services available is one component of a greater cultural and structural change aimed at restructuring the hidden curriculum [5].

### Near-peer and faculty mentorship

As the significance of role models in professional identity formation is well established [19], we suggest tailored mentorship programs that increase exposure to role models with similar lived experiences to empower trainees to embrace their unique experiences. This approach is supported by emerging evidence regarding gender disparity in surgery, which suggests that a paucity of same-gender role models prevents women from conceiving of a career in surgery [20, 21]. Mentorship programs for staff have also been identified as an effective way to increase recruitment and retention of underrepresented minorities in medical school faculties [22, 23], which could in turn increase the availability of diverse role models.

### Formal curricula

Formal teaching that acknowledges students’ diverse experiences and draws on those experiences as forms of expertise will better prepare all students to treat the diverse populations they serve [24, 25]. In their work on underrepresented minority medical students, Rumala and Cason [26] illustrate the value of formally collaborating with student minority organizations in improving peer support and recruitment programs. The curriculum, with support from senior leadership in the medical institution, can therefore empower students to value and learn from their diverse positionalities as assets to their professional identity formation [27].

In closing, we are grateful to medical institutions for their efforts to increase diversity in medical school classes. However, the next important step is to support the more personal influences on our ‘figured worlds’ that bear real-world significance. Empowering students to integrate their pre-medical backgrounds into their developing identity is crucial not only to improving the process of professional identity formation for diverse students, but also to nurturing empathic and insightful physicians uniquely suited to respond to the plights of their patients.
